# Affective norms for 501 Chinese words from three emotional dimensions rated by depressive disorder patients

**DOI:** 10.3389/fpsyt.2024.1309501

**Published:** 2024-02-26

**Authors:** Xinyue Xu, Fei An, Shengjun Wu, Hui Wang, Qi Kang, Ying Wang, Ting Zhu, Bing Zhang, Wei Huang, Xufeng Liu, Xiuchao Wang

**Affiliations:** ^1^ Department of Military Medical Psychology, Air Force Medical University, Xi'an, China; ^2^ Department of Clinical Psychology, Dongguan Seventh People’s Hospital, Dongguan, China; ^3^ Center for Psychological Crisis Intervention, the 904th Hospital of the Joint Logistics Support Unit, Changzhou, China; ^4^ Department of Psychosomatic Medicine, Xi’an International Medical Center, Xi'an, China; ^5^ Xinfeng Psychiatric Hospital, Xi ‘an Ninth Hospital, Xi'an, China; ^6^ Department of Medical Psychology, the 984th Hospital of the Joint Logistics Support Unit, Beijing, China; ^7^ Department of Psychiatry, the 923th Hospital of the Joint Logistics Support Unit, Nanning, China

**Keywords:** affective norms, Chinese words, depression, word evaluation, social media

## Abstract

**Introduction:**

Emotional words are often used as stimulus material to explore the cognitive and emotional characteristics of individuals with depressive disorder, while normal individuals mostly rate the scores of affective words. Given that individuals with depressive disorder exhibit a negative cognitive bias, it is possible that their depressive state could influence the ratings of affective words. To enhance the validity of the stimulus material, we specifically recruited patients with depression to provide these ratings.

**Methods:**

This study provided subjective ratings for 501 Chinese affective norms, incorporating 167 negative words selected from depressive disorder patients’ Sino Weibo blogs, and 167 neutral words and 167 positive words selected from the Chinese Affective Word System. The norms are based on the assessments made by 91 patients with depressive disorder and 92 normal individuals, by using the paper-and-pencil quiz on a 9-point scale.

**Results:**

Regardless of the group, the results show high reliability and validity. We identified group differences in three dimensions: valence, arousal, and self-relevance: the depression group rated negative words higher, but positive and neutral words lower than the normal control group.

**Conclusion:**

The emotional perception affected the individual’s perception of words, to some extent, this database expanded the ratings and provided a reference for exploring norms for individuals with different emotional states.

## Introduction

As reported, depression currently accounts for 4.3% of the global burden of disease, and is expected to be the leading cause of disease burden in high-income countries by 2030 (1). People suffering from depression may experience anhedonia, concentration difficulties, disordered eating and sleeping patterns, feelings of worthless and persistent sadness. Due to the profound negative effects, that depression has on people’s lives, it is critical to diagnose and treat it as early as possible. In general, detecting depression requires thorough and detailed psychological testing by experienced psychiatrists according to acknowledged diagnostic criteria, including interviews, questionnaires, self-reports or testimony from friends and relatives (2). However, people who suffer from depression do not always visit clinics to ask for professional help in the early stage of this disease (3). Due to a lack of authentic social interaction and the fear of being judged, individuals with depressive disorder may use social media networking to express their thoughts and feelings with people similar to themselves (4). Social media platforms such as Twitter, Facebook, Reddit and Instagram offer a virtual community network where people of various demographic backgrounds share sentiments, exchange information, and provide mutual support for common conditions (5).

Numerous studies from the literature have demonstrated that social media can be an important avenue for predicting depression (6–10). Surveillance of the online content and users’ posting activity has been proposed as a complementary or alternative precision tool for the early detection of depression markers (5). In most studies, research objects include posts and photographs shared on social media by users with depressive disorder (11), and the posts are the main object between them. Ophir et al. (12) found that adolescents who explicitly expressed distress in their posts had a higher Beck Depression Inventory II (BDI-II) score than those who did not. Settanni & Marengo (13) found that the presence of depression was positively correlated with the expression of negative emotions on Facebook. Another study found that individuals with depressive disorder tend to use more first-person singular pronouns, such as my and me. In addition, words related to sad mood (tears, cry, pain), loneliness (miss, much, baby), and hostility (hate, ugh, fuckin) were also observed (8). Similarly, Brockmeyer et al. (14) revealed that the use of first-person singular pronouns significantly predicted depressive symptoms approximately 8 months later, even after controlling for depressive symptoms at baseline or discharge. In addition, Gaikar, Chavan, Indore, & Shedge (15) used an SVM(support vector machine) classifier to detect depression-related words and sentences and verified types of depression from those identified words. Cacheda, Fernandez, Novoa, & Carneiro (16) identified several distinctive features of posting activity associated with the onset of depression, such as diurnal cycles, increased negative emotions, decreased social interactions, increased self-focus, and increased mentions of depression-related terms. Leis, Ronzano, Mayer, & Furlong (17) found that the proportion of negative words in depressive user datasets was significantly higher than those in the control dataset. The proportion of polarity of tweets from the two groups was also significantly different, the depressive tweet dataset showed more negative polarity, but the control tweet dataset showed more positive polarity. Based on the above studies, language, especially emotional words, is an important marker in terms of recognizing depression at early stages. In addition, different emotional words also elicited different emotional experiences, which may affect people’s cognition, such as attention (18–20) and memory (21–24). Nonetheless, emotional words used in these studies were standardized because researchers established norms for the emotional characteristics of words. The aim of this study was to explore the characteristics of emotional words in depressed individuals.

According to dimensional models, emotional stimuli are often characterized by two dimensions: (1) valence (ranging from negative to positive) and (2) arousal (ranging from calm to exciting) (25). In addition, dominance or control was another dimension, that was used to discriminate one emotion from another. However, dominance has not been examined to the same extent as valence and arousal in the affective literature, so it has not been widely referenced in the literature (26). The Affective Norms for English Words (ANEW) database was published in 1999 by Bradley and Lang, and has become the authoritative emotional word database (27). Since then, the ANEW has been adapted to other languages, such as Polish (28), Spanish (29) (30), German (31) and Portuguese (32). In view of this, we found that the meaning of words might change under different language circumstances.

The present study aimed to provide a set of depression-related affective norms rated by depressive disorder patients. The cognitive model of depression posits that depression symptoms are maintained by negatively biased cognition, particularly negative cognition about the self. Furthermore, this kind of negative cognition had consistency, namely, it showed negative cognition regarding everything. This range allowed us to determine whether evaluations of affective norms change with depressive symptoms. Specifically, we collected data for the affective dimensions of valence, arousal and self-relevance, and calculated their reliability and validity. We then verified the U-shaped relationship between valence and arousal that was inferred in previous studies (33). Finally, we compared the differences in ratings between the depression group and the normal control group. Consistent with past research (33), our hypotheses were as follows: First, valence and arousal exhibit a U-shaped trend, and self-relevance shows a negative correlation with valence, but a positive correlation with arousal in the depression group. Second, in all dimensions—valence, arousal, and self-relevance—in terms of negative words, the scores of the depression group were higher than those of the normal control group, while the opposite was true for neutral words and positive words.

## Method

### Participants

Ninety-nine depressive disorder patients (M=26.15 years, SD=7.16 years, range=18-51 years, 26 females) were recruited from the psychology outpatient department and inpatient ward of Xi’an International Medical Center, Xi’an Ninth Hospital, the 904th Hospital of the Joint Logistics Support Unit, the 984th Hospital of the Joint Logistics Support Unit and the 923th Hospital of the Joint Logistics Support Unit. Ninety-two normal control participants(M=24.47 years, SD=8.20 years, range =18-55 years, 25 females)were recruited by hospital advertisements. All participants were right-handed and had normal perceptual and verbal communication skills.

To ensure that all participants met the requirements, the participants must fill out two questionnaires, one for the self-rated Beck Depression Inventory (BDI) (34) and the other for the other-rated Hamilton Depression Inventory (HAMD) (35). The depression group was diagnosed by a physician meeting depressive diagnostic criteria in the Diagnostic and Statistical Manual of Mental Disorders (36), meanwhile, their total HAMD score was higher than 17 points, and the total BDI score was higher than 28 points. The total HAMD score of the normal control group was less than 7 points, and the total BDI score was less than 14 points. This study was approved by the Clinical Trial Ethics Committee of Xijing Hospital (Lot No: KY 20222089-F-2). All participants (or their guardians) signed a written informed consent prior to starting the study.

Four depressive disorder patients were removed because their proportion of identical answers in the questionnaire was greater than 85%, and four depressive disorder patients were removed because they did not complete all questions due to their emotional conditions (37). All normal control group participants completed the entire questionnaire with less than 85% of the same answers. Therefore, the final sample consisted of ninety-one depressive disorder patients (*M*= 26.02 years, *SD* = 6.79 years, range = 18–51 years, 23 females) and ninety-two normal control participants(*M*=24.47 years, *SD*=8.20 years, range =18-55 years, 25 females). There were no differences between the two groups of participants in terms of gender (*χ* ^2^=0.09, *p*=0.770), age (*t*=-1.34, *p*=0.183) and education level (*χ* ^2^ = 2.14, *p*=0.343), but the depression group’s total BDI score and total HAMD score were both significantly higher than those of normal control participants (*t*=35.41, *p*<0.001; *t*=42.27, *p*<0.001). Sample characteristics are displayed in **Table 1**.

**Table 1:** Sample characteristics of the depression group (n = 91) and normal control group (n = 92).

		Depression Group (*M* ± *SD*)	Normal Control Group (*M* ± *SD*)	Statistical values		*p*
Age (in years)		25.98 ± 6.85	24.48 ± 8.25	*t*=-1.34		0.183
BDI total score		37.27 ± 8.28	3.96 ± 3.49	*t*=35.41		<0.001
HAMD total score		22.42 ± 4.69	1.03 ± 1.14	*t*=42.27		<0.001
Sex	male	68	67	*χ* ^2^ = 0.09		0.770
	female	23	25			
Education level	middle school and below	6	2			
	high school	20	21	*χ* ^2^ = 2.14		0.343
	university and above	65	69			

### Materials and procedure

This study focused on depressive disorder patients’ blogs released in Sina Weibo (https://weibo.com/). Depressive users must meet the following conditions simultaneously. Their responses must contain sentences which expressed “I was diagnosed with depression” or “I have depression” or “I was depressed”. In addition, their blogs always expressed negative feelings, and they had less than 500 followers. First, the author manually located depressive users in the Depression Super Topic. The researcher then used a homemade crawler software program to crawl the retrieved information related to the users with depressive disorder in this section and performed data cleaning. Following this, 8 psychology graduate students were recruited to conduct manual annotation and qualitative analysis of the tweets. We then invited 3 clinical psychiatrists to discuss the data and to identify depressive features in the posts. Finally, the study counted word frequencies of depression-related tests and deleted word frequencies that occurred less than 5 times. In the end, 167 negative words were saved. To match these words and test the validity, this study selected another 167 positive words (*M*=7.19, *SD*=0.28, range=6.39-7.82) and 167 neutral words (*M*=5.36, *SD*=0.64, range=3.88-6.55) from the Chinese Affective Word System (38).

Three dimensions of 501 words were used to develop three paper questionnaires (valence, arousal and self-relevance), and all participants completed the paper questionnaire in no more than two days. For unspecified reasons, three depressive disorder patients did not rate positive words, and one did not rate negative words. These data were obtained from December 2021 to July 2022.

At the beginning of the experiment, the participants completed an informed consent form, demographic questions (age, gender and education) and the HAMD and BDI inventories. Each participant was given instructions for responding to the three dimensions before starting the rating procedure, and each received a pen to record their responses. The instructions were cited from previous Chinese normative studies (37–39). Before the test, the researcher reviewed the instructions with the participants. When it was determined that each understood the directions, the participants began the test and were reassured that there were no right or wrong answers. Rather, they were asked to respond to each item based on their first instinct. The paper-and-pencil quiz used a nine-point scale, and all participants drew a tick on the corresponding number based on their own instinct.

## Results

The results contained three parts. First, we examined the reliability by calculating the internal consistency coefficient and comparing to the ratings of previous studies to confirm research validity. Second, we investigated the relationship between the dimensions in both cohorts. Finally, we compared the differences in the ratings of words across the two groups. All correlations reported in this study were Pearson correlations.

### Descriptive statistics

**Figure 1:**
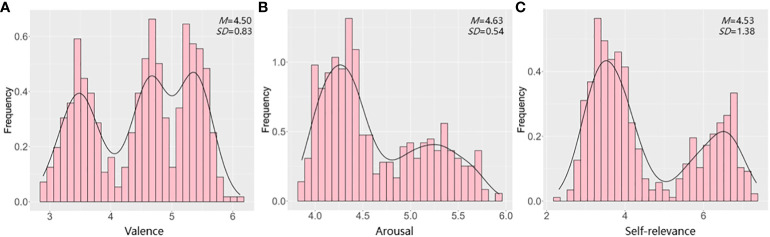
Distributions and descriptive statistics of 501 affective words. **(A)** distributions and descriptive statistics of valence ratings. **(B)** distributions and descriptive statistics of arousal ratings. **(C)** distributions and descriptive statistics of self-relevance ratings.

The distributions and descriptive statistics for valence, arousal and self-relevance ratings were displayed in **Figure 1**. Consistent with prior reports (37, 40), the three distributions deviated significantly from a normal distribution. The distribution of valence ratings was negatively skewed (G1=-0.24). It was observed that 66.07% of 501 words were rated under the middle of the valence rating scale (score of 5). The arousal dimension was characterized by a positive skew (G1=0.61), and we observed that 71.26% of 501 words were rated under the middle of the valence rating scale (score of 5). Consistent with arousal, the distribution of self-relevance ratings was also positively skewed (G1=0.57). It was observed that 65.87% of 501 words were rated under the middle of the valence rating scale (score of 5).

### Reliability of valence, arousal and self-relevance ratings

To explore the reliability of our methods, we calculated the internal consistency coefficient (ICC) of three dimensions ratings (valence, arousal and self-relevance) by depressive disorder patients. The result of Cronbach’s alpha demonstrated that the present database had high internal consistency (α in **Table 2**). In addition, we randomly divided depressive disorder patients into two subgroups and calculated their mean ratings for each word. Then, we calculated the Pearson correlation coefficient between the two subgroups. We found that the correlations between the two subgroups were high for valence (*r*=0.931, *p*<0.001), arousal (*r*=0.847, *p*<0.001) and self- relevance (*r*=0.965, *p*<0.001).

**Table 2:** Reliability for three affective dimensions.

	Valence	Arousal	Self-relevance
Cronbach’s alpha	0.992	0.996	0.991
Pearson correlation coefficient	0.931^***^	0.847^***^	0.965^***^

^***^p<0.001.

### Consistency of the present and previous ratings

To further explore the validity of these affective norms, we compared current scores with those reported in a previous study (38). This current study’s corpus contained 378 words from a prior study. Pearson correlations were calculated between scores of overlapping words collected from the present and previous norms. Pearson correlation between the overlapping words ratings by depressive disorder patients included in the present study and in Wang was significant in valence (*r*=0.928, *p*<0.001) and arousal (*r*=0.378, *p*<0.001). Meanwhile, we calculated the Pearson correlation of scores of the overlapping words for valence and arousal between normal control group individuals and Wang. The results showed that the scores were also significantly in valence (*r*=0.965, *p*<0.001) and arousal (*r*=0.751, *p*<0.001). These results revealed that the participants views of the present study aligned substantially with those in Wang’s research.

### Relationship among dimensions in depressive disorder patients

**Figure 2:**
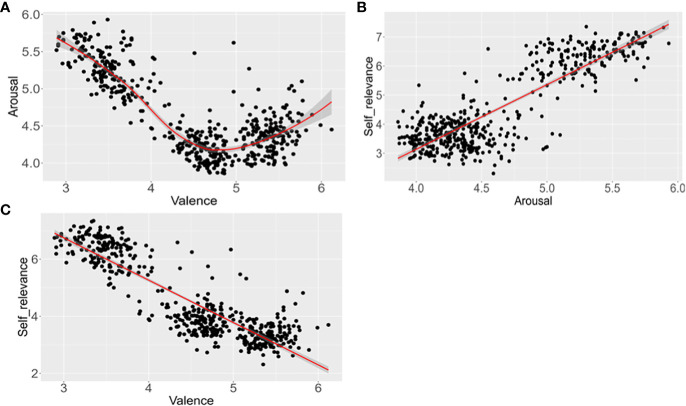
Distribution of the mean ratings for the 501 words among different affective dimensions. **(A)** distribution of the mean ratings in the valence and arousal variables. **(B)** distribution of the mean ratings in the arousal and self-relevance variables. **(C)** distribution of the mean ratings in the valence and self-relevance variables.

The position of mean word ratings in a two-dimensional affective space defined by valence, arousal and self-relevance was demonstrated in **Figure 2**. To explore the relationship between the valence and arousal dimensions, regression analysis was carried out. We calculated both the linear function with mean valence as an independent factor and mean arousal as a dependent factor and quadratic functions with mean valence and its square as independent factors, and mean arousal as a dependent factor. The results showed that there was a typical quadratic U-shaped relation between valence and arousal [*R*=0.874, *F* (2, 498) = 806.517, *p*<0.001]. The quadratic model seemed to be more suitable because it explained 75.8% of the variance, while the linear model explained 56.0% of the variance. This result demonstrated that increased positive or negative words would be more arousing than valence-neutral words. This phenomenon was consistent with previous studies (41–43).

In addition, we calculated the Pearson correlation coefficient and used regression analyses to explore the relationship between valence and self-relevance dimensions, with mean valence as an independent factor and mean self-relevance as a dependent factor. Valence was negatively related to self-relevance (*r*=-0.887, *p*<0.01), and there was a linear relationship between the valence and self-relevance dimensions, *R*=0.887, *F* (1, 499) = 1835.872, *p*<0.001. This linear model was appropriate as it explained 78.6% of the variance. This result was consistent with the negative cognitive characteristics of patients with depression.

Finally, we calculated the Pearson correlation coefficient and used regression analyses to explore the relationship between arousal and self-relevance dimensions, with mean arousal as an independent factor and mean self-relevance as a dependent factor. Arousal was related to self-relevance (*r*=0.860, *p*<0.01), and there was a linear relationship between the arousal and self-relevance dimensions, *R*=0.860, *F* (1, 499) = 1415.385, *p*<0.001. The linear model below explains 73.9% of the variance.

### Group differences in the valence, arousal and self-relevance of affective norms

**Figure 3:**
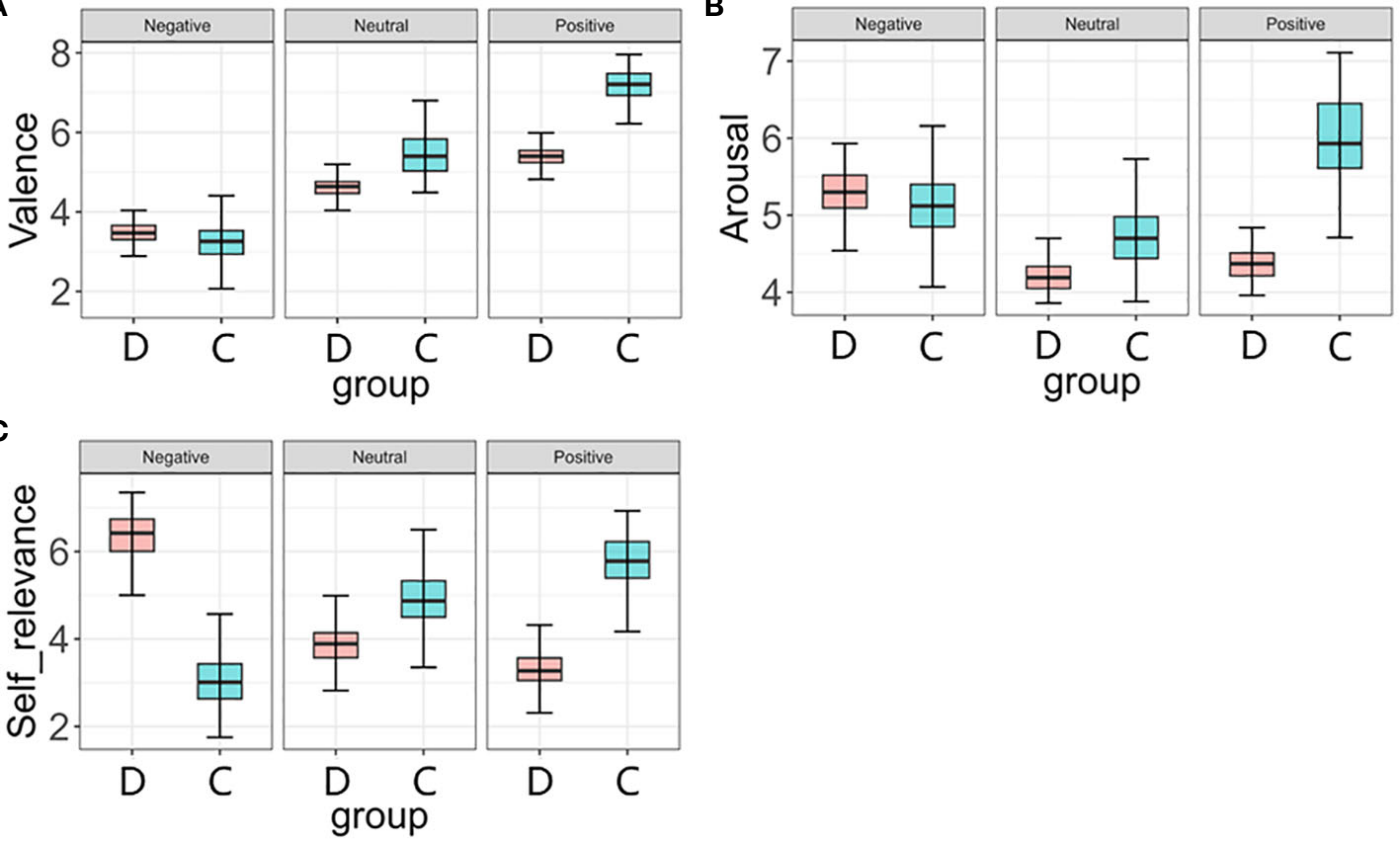
Mean rating for control normal group and depression group in different lexical properties words. **(A)** mean ratings of valence; **(B)** mean rating of arousal; **(C)** mean ratings of self-relevance; C, Control normal group; D, Depression group.

**Table 3** demonstrates descriptive statistics of valence, arousal, and self-relevance ratings for 501 affective norms by depression group and normal control group. For each dimension, we conducted a repeated-measures ANOVA with one between-subject factor: group (depression group vs. control normal group) and one within-subject factor: lexical property (negative words vs. neutral words vs. positive words). In valence, repeated-measures ANOVA revealed main effects of group [*F*(1,332)=1265.52, *p*<0.001, $\eta_{p}^{2}$ = 0.998], lexical property [*F*(2,332)=5117.38, *p*<0.001, $\eta_{p}^{2}$ =0.939] and the interaction effect between group and lexical property [*F*(2,332)=635.90, *p*<0.001, $\eta_{p}^{2}$ =0.657]. Because the interaction effect was significant, we further conducted a simple effect test. The results showed that ratings of the depression group were significantly higher than the normal control group in negative words [*F*(1,332)=38.66, *p*<0.001, $\eta_{p}^{2}$ =0.104], but lower in neutral words [*F*(1,332)=366.72, *p*<0.001, $\eta_{p}^{2}$ =0.525] and positive words [*F*(1,332)=2886.05, *p*<0.001, $\eta_{p}^{2}$ =0.897]. In arousal, repeated-measures ANOVA revealed main effects of group [*F*(1,332)=594.01, *p*<0.001, $\eta_{p}^{2}$ =0.641], lexical property [*F*(2,332)=509.96, *p*<0.001, $\eta_{p}^{2}$ =0.606] and the interaction effect between group and lexical property [*F*(2,332)=588.21, *p*<0.001, $\eta_{p}^{2}$ =0.639]. We further conducted a simple effect test due to the significant interaction effect. The results showed that ratings of the depression group were significantly higher than the normal control group in negative words [*F*(1,332)=21.05, *p*<0.001, $\eta_{p}^{2}$ =0.060], but lower in neutral words [*F*(1,332)=208.35, *p*<0.001, $\eta_{p}^{2}$ =0.386] and positive words [*F*(1,332)=1221.94, *p*<0.001, $\eta_{p}^{2}$ =0.786]. In self-relevance, repeated-measures ANOVA revealed main effects of lexical property [*F*(2,332)=23.67, *p*<0.001, $\eta_{p}^{2}$ =0.067] and the interaction effect between group and lexical property [*F*(2,332)=2185.21, *p*<0.001, $\eta_{p}^{2}$ =0.868]. No significant main effect of group was found [*F*(1,332)=1.09, *p*=0.297, $\eta_{p}^{2}$ =0.003]. The result of simple effect test demonstrated that ratings of the depression group were significantly higher than the normal control group in negative words [*F*(1,332)=2609.74, *p*<0.001, $\eta_{p}^{2}$ =0.887], but lower in neutral words [*F*(1,332)=238.67, *p*<0.001, $\eta_{p}^{2}$ =0.418] and positive words [*F*(1,332)=1679.33, *p*<0.001, $\eta_{p}^{2}$ =0.836]. The mean word ratings between two groups in different lexical properties were depicted in **Figure 3**.

**Table 3:** Descriptive statistics in valence, arousal, and self-relevance ratings for 501 affective norms.

	Depression Group					Normal Control Group				
	Mean	SD	Min	Max	Range	Mean	SD	Min	Max	Range
Valence	4.50	0.83	2.89	6.12	3.23	5.29	1.70	1.64	7.96	6.32
Arousal	4.63	0.53	3.86	5.93	2.07	5.27	0.69	3.88	7.11	3.23
Self-relevance	4.53	1.38	2.31	7.35	5.04	4.57	1.29	1.75	7.50	5.75

C, Control normal group; D, Depression group.

## Discussion

In this study, we explored the relationships among valence, arousal and self-relevance. The results are shown below. First, we found that there was a typical quadratic U-shaped relation between valence and arousal, which was consistent with previous studies, such as Chinese (39), Spanish (44), English (45), Croatian (43) and German (31). The more emotionally charged the word, the higher the arousal level it expressed. Second, a strong negative correlation was observed between valence and self-relevance, depressive disorder patients rated negative words as most relevant to them, followed by neutral words, with positive words being the least relevant. This phenomenon reflected the emotional characteristics of depressive disorder patients. According to the Diagnostic and Statistical Manual of Mental Disorders (DSM), depressed mood or loss of interest or pleasure in activities of daily living are core symptoms in depressive disorder patients (36). According to the emotional congruence effect, people are more likely to select and process stimuli that are equal to their emotional state, demonstrating an initiating effect on a particular emotion (46). Therefore, it was natural for those people to rate negative words as more relevant to themselves. It also illustrated the effectiveness of the words, that were selected from depressive disorder patients’ social media posts. Third, we observed a strong positive correlation between self-relevance and arousal, the higher the level of self-relevance was, the higher the arousal level. Because self-relevant information was more easily captured and individuals were more sensitive to self-relevant stimuli (47), the depression group showed higher arousal to negative words than positive words.

Analysis of the valence, arousal and self-relevance ratings between the depressive group and the normal control group revealed several results. In summary, the depressive group scored significantly higher than the normal control group on the dimension of negative words and significantly lower on the dimensions of neutral and positive words. Regarding valence, the two groups showed the same trend of scoring on words, and ratings on negative words were lower than those on neutral words and lower than those on positive words. Regarding arousal, the normal control group scored neutral words lower than negative words and lower than positive words, but the depression group scored neutral words lower than positive words and lower than negative words. Regarding self-relevance, the normal control group scored negative words lower than neutral words and lower than positive words, while the depression group scored positive words lower than neutral words and lower than negative words. Mood congruence implies that the efficiency of memory processing is biased by the congruence between an existing mood and the affective tone of the material mood (48). Depressive disorder patients tended to attribute negative emotions to neutral stimuli rather than positive emotions, furthermore, their preferential processing of emotionally congruent information in the environment may enhance the recognition of negative emotional words over positive emotional words relative to individuals who are not depressed (49). Therefore, the depression group rated neutral and positive words significantly lower than the normal control group on both dimensions. However, on the valence dimension, the depression group rated the negative words significantly higher than the normal control group, which seemed to contradict the results of mindfulness consistency. The reason for this might lie in the fact that participants in the depression group developed sensory adaptation to negative information. Perceptual systems have the ability to adapt to sustained stimulation, when sensory adaptation occurs, their sensitivities change, in that a longer exposure to a particular stimulus alters judgments about the stimulus presented afterward (50). Because depressive disorder patients had been under negative emotions for a long time, they gradually developed sensory adaptation to negative emotions and words expressing sad feelings, thus raising the sensory threshold for negative words, resulting in higher ratings of negative words in depressive disorder patients than in normal control participants. Stimulus-specific adaptation is thought to be a common phenomenon, that has been studied extensively in both mammals and insects (51).

Our study revealed a distinct variation in words evaluation between patients diagnosed with depressive disorder and healthy individuals, particularly concerning negative words. This observation emphasized the representativeness and applicability of the negative words chosen based on data from Sina Weibo. In the era of burgeoning information technology, social media platforms, such as Sina Weibo, have emerged as significant arenas for individuals suffering from depressive disorders in China to articulate their thoughts, sentiments, and psychological states. These platforms served as a valuable repository of linguistic data, and the negative words derived from this information demonstrated a high level of ecological validity. In light of their substantial ecological validity, these negative words could be applied in a multitude of scenarios. They could serve as crucial indicators for network monitoring when amalgamated with machine learning approaches. In addition, they could support individuals in their everyday decision-making, thereby promoting the early detection of depressive symptoms.

## Limitations and future directions

There were some potential limitations in the present study. First, the participants included in our study were not sufficient for conducting age analysis. In future research, we plan to include participants from different age groups to explore the relationship between age and scores in various dimensions. Second, due to the prevalence of negative words in the Sino Weibo blogs of individuals with depressive disorder, we exclusively selected negative words for this study. In future research, we aim to select positive and neutral words from the Sino Weibo blogs of individuals without depression to use as experimental stimuli and further explored the differences in ratings between groups for positive and neutral words.

## Conclusion

The present research provided preliminary evidence that individuals with depressive disorder, when compared to normal participants, displayed abnormal ratings in valence, arousal and self-relevance dimensions. In summary, our findings indicated that individuals with depressive disorder showed deficits in emotional word processing, and the present research provided a valuable tool for screening depressive patients in clinical settings.

## Data availability statement

The raw data supporting the conclusions of this article will be made available by the authors, without undue reservation.

## Ethics statement

This study was approved by the Clinical Trial Ethics Committee of Xijing Hospital (Lot No: KY20222089-F-2). We have stated in the manuscript. The present study do not included potentially identifiable images.

## Author contributions

XX: Writing – original draft, Writing – review & editing, Conceptualization, Methodology. FA: Writing – original draft, Writing – review & editing, Conceptualization, Methodology. SW: Conceptualization, Methodology, Writing – review & editing. HW: Conceptualization, Methodology, Writing – review & editing. QK: Data curation, Writing – review & editing. YW: Data curation, Writing – review & editing. TZ: Data curation, Writing – review & editing. BZ: Data curation, Writing – review & editing. WH: Data curation, Writing – review & editing. XL: Conceptualization, Funding acquisition, Project administration, Methodology, Writing – review & editing. XW: Conceptualization, Methodology, Funding acquisition, Project administration, Writing – review & editing.

## References

[1] Mathers CD Loncar D. Projections of global mortality and burden of disease from 2002 to 2030. PloS Med. (2006) 3:e442. doi: 10.1371/journal.pmed.0030442 17132052 PMC1664601

[2] Zogan H Razzak I Wang X Jameel S Xu G. Explainable depression detection with multi-aspect features using a hybrid deep learning model on social media. World Wide Web. (2022) 25:281–304. doi: 10.1007/s11280-021-00992-2 35106059 PMC8795347

[3] Zou ML Li MX Cho V. Depression and disclosure behavior via social media: A study of university students in China. Heliyon. (2020) 6:e03368. doi: 10.1016/j.heliyon.2020.e03368 32099917 PMC7031301

[4] Gupta S Goel L Singh A Prasad A Ullah MA. Psychological analysis for depression detection from social networking sites. Comput Intell Neurosci. (2022) 2022:4395358. doi: 10.1155/2022/4395358 35432513 PMC9007657

[5] Owusu PN Reininghaus U Koppe G Bärnighausen I. D.-M. T. Artificial intelligence applications in social media for depression screening: A systematic review protocol for content validity processes. PloS One. (2021) 16:e0259499. doi: 10.1371/journal.pone.0259499 34748571 PMC8575242

[6] Floridi BDML. The ethics of big data: current and foreseeable issues in biomedical contexts. Sci Eng Ethics. (2016) 22:303–41. doi: 10.1007/s11948-015-9652-2 26002496

[7] Reece AG Reagan AJ Lix KLM Dodds PS. Forecasting the onset and course of mental illness with Twitter data. Sci Rep. (2017) 7:13006. doi: 10.1038/s41598-017-12961-9 29021528 PMC5636873

[8] Eichstaedt JC Smith RJ Merchant RM Ungar LH. Facebook language predicts depression in medical records. Proc Natl Acad Sci USA. (2018) 115:11203–8. doi: 10.1073/pnas.1802331115 PMC6217418 30322910

[9] Merchant RM Asch DA Crutchley P Ungar LH. Evaluating the predictability of medical conditions from social media posts. PloS One. (2019) 14:e0215476. doi: 10.1371/journal.pone.0215476 31206534 PMC6576767

[10] Chiong R Budhi GS Dhakal S Chiong F. A textual-based featuring approach for depression detection using machine learning classifiers and social media texts. Comput Biol Med. (2021) 135:104499. doi: 10.1016/j.compbiomed.2021.104499 34174760

[11] Kim J Uddin ZA Lee Y Nasri F Gill H. A Systematic review of the validity of screening depression through Facebook, Twitter, Instagram, and Snapchat. J Affect Disord. (2021) 286:360–9. doi: 10.1016/j.jad.2020.08.091 33691948

[12] Ophir Y Asterhan CSC Schwarz BB. The digital footprints of adolescent depression, social rejection and victimization of bullying on Facebook. Comput Hum Behav. (2019) 91:62–71. doi: 10.1016/j.chb.2018.09.025

[13] Settanni M Marengo D. Sharing feelings online: studying emotional well-being via automated text analysis of Facebook posts. Front Psychol. (2015) 6:1045. doi: 10.3389/fpsyg.2015.01045 26257692 PMC4512028

[14] Brockmeyer JZT Hunn M Schauenburg H. First-person pronoun use in spoken language as a predictor of future depressive symptoms: preliminary evidence from a clinical sample of depressed patients. Clin Psychol Psychother. (2017) 24:384–91. doi: 10.1002/cpp.2006 26818665

[15] Gaikar M Chavan J Indore K Shedge R. Depression detection and prevention system by analysing tweets. Soc Sci Res Network. (2019). doi: 10.2139/ssrn.3358809

[16] Cacheda F Fernandez D Novoa FJ Carneiro V. Early detection of depression: social network analysis and random forest techniques. J Med Internet Res. (2019) 21:e12554. doi: 10.2196/12554 31199323 PMC6598420

[17] Leis A Ronzano F Mayer MA Furlong LI. Detecting signs of depression in tweets in spanish: behavioral and linguistic analysis. J Med Internet Res. (2019) 21:e14199. doi: 10.2196/14199 31250832 PMC6620890

[18] Aquino JM Arnell KM. Attention and the processing of emotional words: dissociating effects of arousal. Psychonomic Bull Rev. (2007) 14:430–5. doi: 10.3758/bf03194084 17874583

[19] Thapar A Rouder JN. Aging and recognition memory for emotional words: a bias account. Psychonomic Bull Rev. (2009) 16:699–704. doi: 10.3758/pbr.16.4.699 19648455

[20] Fraga I, Parmentier FBR Leiva A Ferré P. Distraction by deviant sounds: disgusting and neutral words capture attention to the same extent. psychol Res. (2020) 84:1801–14. doi: 10.1007/s00426-019-01192-4 PMC7478951 31053888

[21] Schmidt SR. Memory for emotional words in sentences: the importance of emotional contrast. Cogn Emotion. (2012) 26:1015–35. doi: 10.1080/02699931.2011.631986 22394109

[22] Liu W-H Wang L-Z Zhao S-H Ning Y-P. Anhedonia and emotional word memory in patients with depression. Psychiatry Res. (2012) 200:361–7. doi: 10.1016/j.psychres.2012.07.025 22910474

[23] Adelman JS Estes Z. Emotion and memory: a recognition advantage for positive and negative words independent of arousal. Cognition. (2013) 129:530–5. doi: 10.1016/j.cognition.2013.08.014 24041838

[24] Ferré P Fraga I Comesaña M Sánchez-Casas R. Memory for emotional words: The role of semantic relatedness, encoding task and affective valence. Cogn Emotion. (2015) 29:1401–10. doi: 10.1080/02699931.2014.982515 25435268

[25] Russell JA. A circumplex model of affect. J Pers Soc Psychol. (1980) 39:1161–78. doi: 10.1037/h0077714

[26] Osgood CE. The nature and measurement of meaning. Psychol Bull. (1952) 49:197–237. doi: 10.1037/h0055737 14930159

[27] Hollis G Westbury C. When is best-worst best? A comparison of best-worst scaling, numeric estimation, and rating scales for collection of semantic norms. Behav Res Methods. (2018) 50:115–33. doi: 10.3758/s13428-017-1009-0 29322399

[28] Imbir KK. Affective norms for 1,586 Polish words (ANPW): Duality-of-mind approach. Behav Res Methods. (2015) 47:860–70. doi: 10.3758/s13428-014-0509-4 PMC4545176 25294041

[29] Redondo J Fraga I Padrón I Comesaña M. The Spanish adaptation of ANEW (affective norms for English words). Behav Res Methods. (2007) 39:600–5. doi: 10.3758/bf03193031 17958173

[30] Ferré P Guasch M Moldovan C Sánchez-Casas R. Affective norms for 380 Spanish words belonging to three different semantic categories. Behav Res Methods. (2012) 44:395–403. doi: 10.3758/s13428-011-0165-x 22042646

[31] Schmidtke DS Schröder T Jacobs AM Conrad M. ANGST: affective norms for German sentiment terms, derived from the affective norms for English words. Behav Res Methods. (2014) 46:1108–18. doi: 10.3758/s13428-013-0426-y 24415407

[32] Soares AP Comesaña M Pinheiro AP Simões A. The adaptation of the affective norms for english words (ANEW) for european portuguese. Behav Res Methods. (2012) 44:256–69. doi: 10.3758/s13428-011-0131-7 21751068

[33] Sarli L Justel N. Emotional words in Spanish: Adaptation and cross-cultural differences for the affective norms for English words (ANEW) on a sample of Argentinian adults. Behav Res Methods. (2021). doi: 10.3758/s13428-021-01682-7 34505999

[34] Beck AT Ward CH Mendelson M Mock J. An inventory for measuring depression. Arch Gen Psychiatry. (1961) 4:561–71. doi: 10.1001/archpsyc.1961.01710120031004 13688369

[35] Hamilton M. A rating scale for depression. J Neurol Neurosurg Psychiatry. (1960) 23:56–62. doi: 10.1136/jnnp.23.1.56 14399272 PMC495331

[36] Battle DE. Diagnostic and statistical manual of mental disorders (DSM). Codas. (2013) 25:191–2. doi: 10.1590/s2317-17822013000200017 24413388

[37] Liu P Lu Q Zhang Z Tang J. Age-related differences in affective norms for chinese words (AANC). Front Psychol. (2021) 12:585666. doi: 10.3389/fpsyg.2021.585666 33935850 PMC8082186

[38] Wang Y Zhou L Luo Y. The pilot establishment and evaluation of chinese affective words system. Chin Ment Health J. (2008) 22:608–12. doi: 10.3321/j.issn:1000-6729.2008.08.014

[39] Yao Z Wu J Zhang Y Wang Z. Norms of valence, arousal, concreteness, familiarity, imageability, and context availability for 1,100 Chinese words. Behav Res Methods. (2017) 49:1374–85. doi: 10.3758/s13428-016-0793-2 27553483

[40] Fairfield B Ambrosini E Mammarella N Montefinese M. Affective norms for italian words in older adults: age differences in ratings of valence, arousal and dominance. PloS One. (2017) 12:e0169472. doi: 10.1371/journal.pone.0169472 28046070 PMC5207701

[41] Monnier C Syssau A. Affective norms for French words (FAN). Behav Res Methods. (2014) 46:1128–37. doi: 10.3758/s13428-013-0431-1 24366716

[42] Imbir KK. Affective norms for 4900 polish words reload (ANPW_R): assessments for valence, arousal, dominance, origin, significance, concreteness, imageability and, age of acquisition. Front Psychol. (2016) 7:1081. doi: 10.3389/fpsyg.2016.01081 27486423 PMC4947584

[43] Ćoso B Guasch M Ferré P Hinojosa JA. Affective and concreteness norms for 3,022 Croatian words. Q J Exp Psychol. (2019) 72:2302–12. doi: 10.1177/1747021819834226 30744508

[44] Sabater L Guasch M Ferré P Fraga I. Spanish affective normative data for 1,406 words rated by children and adolescents (SANDchild). Behav Res Methods. (2020) 52(5):1939–50. doi: 10.3758/s13428-020-01377-5 32096105

[45] Montefinese M Ambrosini E Fairfield B Mammarella N. The adaptation of the Affective Norms for English Words (ANEW) for Italian. Behav Res Methods. (2014) 46(3):887–903. doi: 10.3758/s13428-013-0405-3 24150921

[46] Zhuang J. Factors which affect emotion congruent effects. Psychol Sci. (2006) 29:1104–6. doi: 10.16719/j.cnki.1671-6981.2006.05.018

[47] Zhang Q Deng N Jiang X LI W. The time course of self-relevance affecting emotional word processing. Acta Psychologica Sin. (2020) 52:946–57. doi: 10.3724/SP.J.1041.2020.00946

[48] Blaney PH. Affect and memory: A review. Psychol Bull. (1986) 99:229–46. doi: 10.1037//0033-2909.99.2.229. 3515383

[49] Vleet TV Stark-Inbara A Merzenich MM Jordan JT. Biases in processing of mood-congruent facial expressions in depression. Psychiatry Res. (2019) 275:143–8. doi: 10.1016/j.psychres.2019.02.076 PMC6504610 30908978

[50] Witthoft N Sha L Winawer J Kiani R. Sensory and decision-making processes underlying perceptual adaptation. J Vis. (2018) 18:10. doi: 10.1167/18.8.10 PMC6108310 30140892

[51] Whitmire CJ Stanley GB. Rapid sensory adaptation redux: A circuit perspective. Neuron. (2016) 92:298–315. doi: 10.1016/j.neuron.2016.09.046 27764664 PMC5076890

